# Aggressive behavior and severe mental disorders in Prison psychiatry

**DOI:** 10.1192/j.eurpsy.2023.1132

**Published:** 2023-07-19

**Authors:** A. Voulgaris

**Affiliations:** Institute for Sexual Medicine and Forensic Psychiatry, Universitätsklinikum Hamburg Eppendorf, Hamburg, Germany

## Abstract

**Introduction:**

Aggressive behavior can be understood as a compelx and social phenomenon. A number of studies have shown that the risk of aggressive behavior is increased for patients with severe mental disorders such as schizophrenia. Although specialized forensic institutions exist in many countries, most offenders with mental disorders are still found in prison settings what corresponds to international literature suggesting an increased prevalence of mental disorders in prison inmates. Still, data on the specific characteristics of patients demonstrating aggressive behavior in medical and mental health settings is limited, especially for prison environments.

**Objectives:**

The aim of our study was to identify patient characteristics that are potentially associated with aggressive incidents in a prison psychiatric setting.

**Methods:**

In routine documentation in German prisons, specific incidents, as e.g. aggressive behavior are reported through an official reporting system. Analyzing these official reports, we collected all aggressive incidents concerning at the Department of Psychiatry of the Berlin prison hospital between 1997 and 2019. In addition, for each patient acting aggressively, we collected data on an equal number of patients who did not demonstrate this behavior during their hospital stay. For those patients with more than one inpatient treatment period, only the first stay in the prison hospital was included. Furthermore, patients were excluded based on age (younger than 16 or older than 70) or death during treatment. The statistical data was analyzed descriptively.

**Results:**

In total, 225 treatment episodes were included of which in 118 cases violent behavior were documented. The items older age, German citizenship, previous violent crimes (OR = 0,40, 95 % KI: 0,35 – 1,17) and antipsychotic (OR = 0,28, 95 % KI: 0,14 – 0,55) or antidepressant (OR = 0,35, 95 % KI: 0,13 – 0,88) treatment within six months prior to admission had a rather protective effect on the occurrence of aggressive behavior during inpatient treatment. Alcohol (OR = 1,21, 95 % KI: 0,64 – 2,27) and drug use disorders (OR = 2.18, 95 % KI: 1,09 – 4,44) tended to be risk factors for aggressive behavior. Thus, the results in this prison psychiatric population correspond to the risk factors described in the literature.

**Conclusions:**

The reported results point in the direction that optimising the availability of psychopharmacological treatment options and offering specialized treatment for patients with comorbid substance use disorder may lead to the prevention of aggressive behavior in patients with a schizophrenia diagnosis.

**Disclosure of Interest:**

None Declared

